# RETRACTION: miR‐654‐5p Targets HAX‐1 to Regulate the Malignancy Behaviors of Colorectal Cancer Cells

**DOI:** 10.1155/bmri/9875031

**Published:** 2025-12-19

**Authors:** BioMed Research International

RETRACTION: F. Huang, X. Wu, M. Wei, H. Guo, H. Li, Z. Shao, Y. Wu, and J. Pu, “miR‐654‐5p Targets HAX‐1 to Regulate the Malignancy Behaviors of Colorectal Cancer Cells,” *BioMed Research International* 2020, no. 1 (2020): 1‐6, https://doi.org/10.1155/2020/4914707.

The above article, published online on 10 February 2020 in Wiley Online Library (http://wileyonlinelibrary.com), has been retracted by agreement between the authors; the journal Section Editor, Raj P. Kandpal; and John Wiley & Sons Ltd.

The retraction has been agreed following an investigation into concerns initially raised on PubPeer [1], where Figures 1D, 1E, 2C, 3C and 3D show significant overlap with figures in publications by different author groups that involved different targets and cell lines [2, 3]. The authors were unable to provide a satisfactory explanation nor provide their original data. As a result, the data and conclusions of this article are considered unreliable.
